# Supplementary material for: Cell cycle-specific phase separation regulated by protein charge blockiness
Source: Nat Cell Biol. 2022 May 5;24(5):625–32. doi: 10.1038/s41556-022-00903-1 (PMC9106583; doi:10.1038/s41556-022-00903-1)
Supplement: Supplementary file 1 — Supplementary Note [file 41556_2022_903_MOESM1_ESM.pdf]

nature portfolio

https://doi.org/10.1038/s41556-022-00903-1

Supplementary information

Cell cycle-specific phase separation regulated by protein charge blockiness

In the format provided by the
authors and unedited

Supplementary Note

Cell cycle-specific phase separation regulated by protein charge blockiness

Hiroya Yamazaki, Masatoshi Takagi, Hidetaka Kosako, Tatsuya Hirano and Shige H. Yoshimura

Mathematical equations

1. Definition of Degree of Segregation (D_{seg})

$D_{seg} = -\log_{10}(p\text{-value}_{\text{U-test}[p+,\, p-]})$,

, where $p+$ and $p-$ represent the position of positively and negatively charged amino acids along the polypeptide, respectively.

2. Definition of Blockiness of Like Charges (B_{LC})

$$B_{LC} = \left(C_{max\,(+)} + C_{max(-)}\right) * \frac{\sum_{k=1}^{Nd(+,-)} d_k(+,-)}{Nd(+,-)} / \left(\frac{\sum_{k=1}^{Nd(+,+)} d_k(+,+)}{Nd(+,+)} + \frac{\sum_{k=1}^{Nd(-,-)} d_k(-,-)}{Nd(-,-)}\right)$$

, where $C_{max(+)}$ and $C_{max(-)}$ represents the absolute maximum positive and negative values in the charge plot, respectively, $d(+, +)$, $d(-, -)$, $d(+, -)$ represent the distance between a pair of charged residues (+; positive charge, –; negative charge) and $Nd(+, +)$, $Nd(-, -)$, $Nd(+, -)$ represent the number of these pairs.

3. Fitting equation to obtain C_{sat}

$OD_{600} = OD_{max}\, C^n / (C_{sat}^n + C^n)$,

,where C represents the protein concentration.

4. Fitting equation for FRAP assay

$$f(t) = Imax\,\left(1 - \sqrt{\frac{\tau D}{\tau D + \pi t}}\right) + C$$

5. Threshold for analyzing the localization of Ki-67 at the mitotic chromosome periphery

α = (95th percentile of signal - 5th percentile of signal)*0.8
