# Supplementary material for: Cell cycle-specific phase separation regulated by protein charge blockiness
Source: Nat Cell Biol. 2022 May 5;24(5):625–32. doi: 10.1038/s41556-022-00903-1 (PMC9106583; doi:10.1038/s41556-022-00903-1)
Supplement: Supplementary file 2 — Reporting Summary [file 41556_2022_903_MOESM2_ESM.pdf]

nature portfolio

Corresponding author(s): Shige H. Yoshimura

Last updated by author(s): Mar 7, 2022

Reporting Summary

Nature Portfolio wishes to improve the reproducibility of the work that we publish. This form provides structure for consistency and transparency in reporting. For further information on Nature Portfolio policies, see our Editorial Policies and the Editorial Policy Checklist.

Statistics

For all statistical analyses, confirm that the following items are present in the figure legend, table legend, main text, or Methods section.

n/a	Confirmed	
☐	☒	The exact sample size (n) for each experimental group/condition, given as a discrete number and unit of measurement
☐	☒	A statement on whether measurements were taken from distinct samples or whether the same sample was measured repeatedly
☐	☒	The statistical test(s) used AND whether they are one- or two-sided<br>*Only common tests should be described solely by name; describe more complex techniques in the Methods section.*
☒	☐	A description of all covariates tested
☒	☐	A description of any assumptions or corrections, such as tests of normality and adjustment for multiple comparisons
☐	☒	A full description of the statistical parameters including central tendency (e.g. means) or other basic estimates (e.g. regression coefficient) AND variation (e.g. standard deviation) or associated estimates of uncertainty (e.g. confidence intervals)
☐	☒	For null hypothesis testing, the test statistic (e.g. F, t, r) with confidence intervals, effect sizes, degrees of freedom and P value noted<br>*Give P values as exact values whenever suitable.*
☒	☐	For Bayesian analysis, information on the choice of priors and Markov chain Monte Carlo settings
☒	☐	For hierarchical and complex designs, identification of the appropriate level for tests and full reporting of outcomes
☐	☒	Estimates of effect sizes (e.g. Cohen's d, Pearson's r), indicating how they were calculated

Our web collection on statistics for biologists contains articles on many of the points above.

Software and code

Policy information about availability of computer code

Data collection	Fluorescence images were obtained by a confocal laser scanning microscope from Olympus (FV-3000) with an accompanying software, FV-31S (2.3.1.163).<br>Optical density was measured by spectrophotometer from JASCO (V-630) with an accompanying software (Spectra Manager 2.10.1).<br>Circular dichroism spectra were measured by a spectropolarimeter from JASCO (J-805) with an accompanying software (Spectra Manager 1.55.0)
Data analysis	Python 3.7.6, Numpy 1.18.1, Pandas 1.0.1, Matplotlib 3.1.3, Scipy 1.4.1, opencv-python 4.4.0, seaborn 0.10.0, Fiji 2.1.0, MetaMorph 7.8.0.0, OriginPro 9.8.0.200, ClustalOmega 1.2.4

For manuscripts utilizing custom algorithms or software that are central to the research but not yet described in published literature, software must be made available to editors and reviewers. We strongly encourage code deposition in a community repository (e.g. GitHub). See the Nature Portfolio guidelines for submitting code & software for further information.

Data

Policy information about availability of data

All manuscripts must include a data availability statement. This statement should provide the following information, where applicable:

- Accession codes, unique identifiers, or web links for publicly available datasets
- A description of any restrictions on data availability
- For clinical datasets or third party data, please ensure that the statement adheres to our policy

The data that support the findings of this study are available in Source Data file and Supporting Information. All other data supporting the findings of this study are available from the corresponding author on reasonable request.

Amino acid sequences of human Ki-67 and human NPM1 can be obtained from UniProt database (accession number P46013 for Ki-67 and P06748 for NPM1)

Field-specific reporting

Please select the one below that is the best fit for your research. If you are not sure, read the appropriate sections before making your selection.

☒ Life sciences ☐ Behavioural & social sciences ☐ Ecological, evolutionary & environmental sciences

For a reference copy of the document with all sections, see nature.com/documents/nr-reporting-summary-flat.pdf

Life sciences study design

All studies must disclose on these points even when the disclosure is negative.

Sample size	No statistical methods were used to predetermine sample size. Sample sizes were estimated empirically on the basis of pilot experiments and previously performed experiments with similar setup to provide sufficient sample sizes for statistical analysis.
Data exclusions	No data was removed from the analysis with the exception of the image analysis of droplet.<br>For the quantification of droplet, ones on the edge of images and ones with higher eccentricity than criterion (0.7) (to exclude the aggregation) were excluded.
Replication	Experiments were repeated multiple times. Turbidity assay has been performed 3 times, and microscopic observation, gel electrophoresis, western blotting and EMSA have been performed at least twice to check data reproducibility.
Randomization	For droplet assay, sample and measurement order was randomized.<br>The area of microscopic observation was randomly determined.
Blinding	Experiments performed in this article were not blinded and this is consistent with what is published in the field.

Reporting for specific materials, systems and methods

We require information from authors about some types of materials, experimental systems and methods used in many studies. Here, indicate whether each material, system or method listed is relevant to your study. If you are not sure if a list item applies to your research, read the appropriate section before selecting a response.

Materials & experimental systems

n/a	Involved in the study
☐	☒ Antibodies
☐	☒ Eukaryotic cell lines
☒	☐ Palaeontology and archaeology
☒	☐ Animals and other organisms
☒	☐ Human research participants
☒	☐ Clinical data
☒	☐ Dual use research of concern

Methods

n/a	Involved in the study
☒	☐ ChIP-seq
☒	☐ Flow cytometry
☒	☐ MRI-based neuroimaging

Antibodies

Antibodies used	anti-NPM1 antibody (Invitrogen, FC-61991)<br>anti-GFP antibody (MBL, Code No.598)<br>HRP-linked anti-rabbit IgG (GE Healthcare, NA-943)<br>HRP-linked anti-mouse IgG (GE Healthcare, NA-931)
Validation	1. anti-NPM1 antibody<br>According to the manufacturer's data, the anti-NPM1 antibody recognizes human, mouse and rat NPM1 in western blotting, Immunohistochemistry, immunoprecipitation, and ELISA. The specificity to human NPM1 in western blot was also confirmed by using the HeLa (human) cell lysate containing mCherry-tagged human NPM1. This antibody recognized both endogenous and exogenous human NPM1 in western blot analysis.<br><br>2. anti-GFP antibody (MBL, Code No. 598)<br>According to the manufacturers data, the polyclonal anti-GFP antibody can detect GFP from Aequorea Victria and its variants (EBFP, SEBFP, ECFP, SECFP, EGFP, SEGFP, cpSEGFP, EYFP, Venus, cpVenus, R-pericam, and Sapphire) on Western blotting, Immunoprecipitation, Immunocytochemistry and Immunohistochemistry. The specificity to EGFP was also confirmed by using the cell lysate from HeLa (human) cells expressing EGFP.

Eukaryotic cell lines

Policy information about cell lines

Cell line source(s)	HeLa cells were purchased from ATCC. Knock-out line of Ki-67 in HCT116 was established in the previous study (Takagi, M. Biochemistry and Biophysics Reports 22, 100720 (2020) by one of the co-authors, M. Takagi (RIKEN, Japan).
Authentication	Knock-out of Ki-67 in HCT116 cells were authenticated by PCR and DNA sequencing.<br>HeLa and HCT116 cells were authenticated by ATCC.
Mycoplasma contamination	All cell lines were tested negative for mycoplasma contamination.
Commonly misidentified lines<br>(See ICLAC register)	No commonly misidentified lines were used in this study.
