# Supplementary figures and images for: Cell cycle-specific phase separation regulated by protein charge blockiness
Source: Nat Cell Biol. 2022 May 5;24(5):625–32. doi: 10.1038/s41556-022-00903-1 (PMC9106583; doi:10.1038/s41556-022-00903-1)

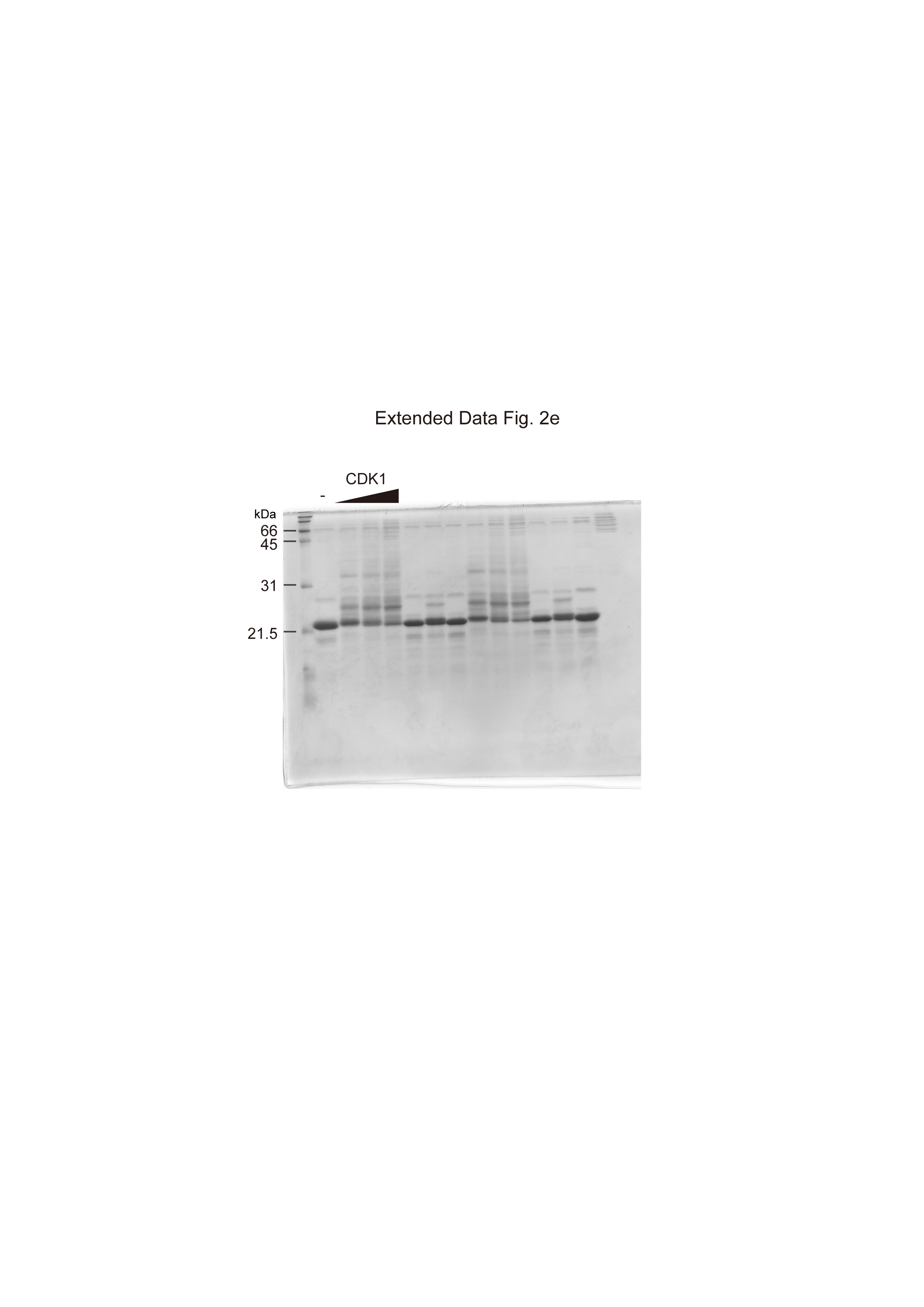

Supplement: Source Data Extended Data Fig. 2 — Unprocessed gel [file 41556_2022_903_MOESM8_ESM.tif]

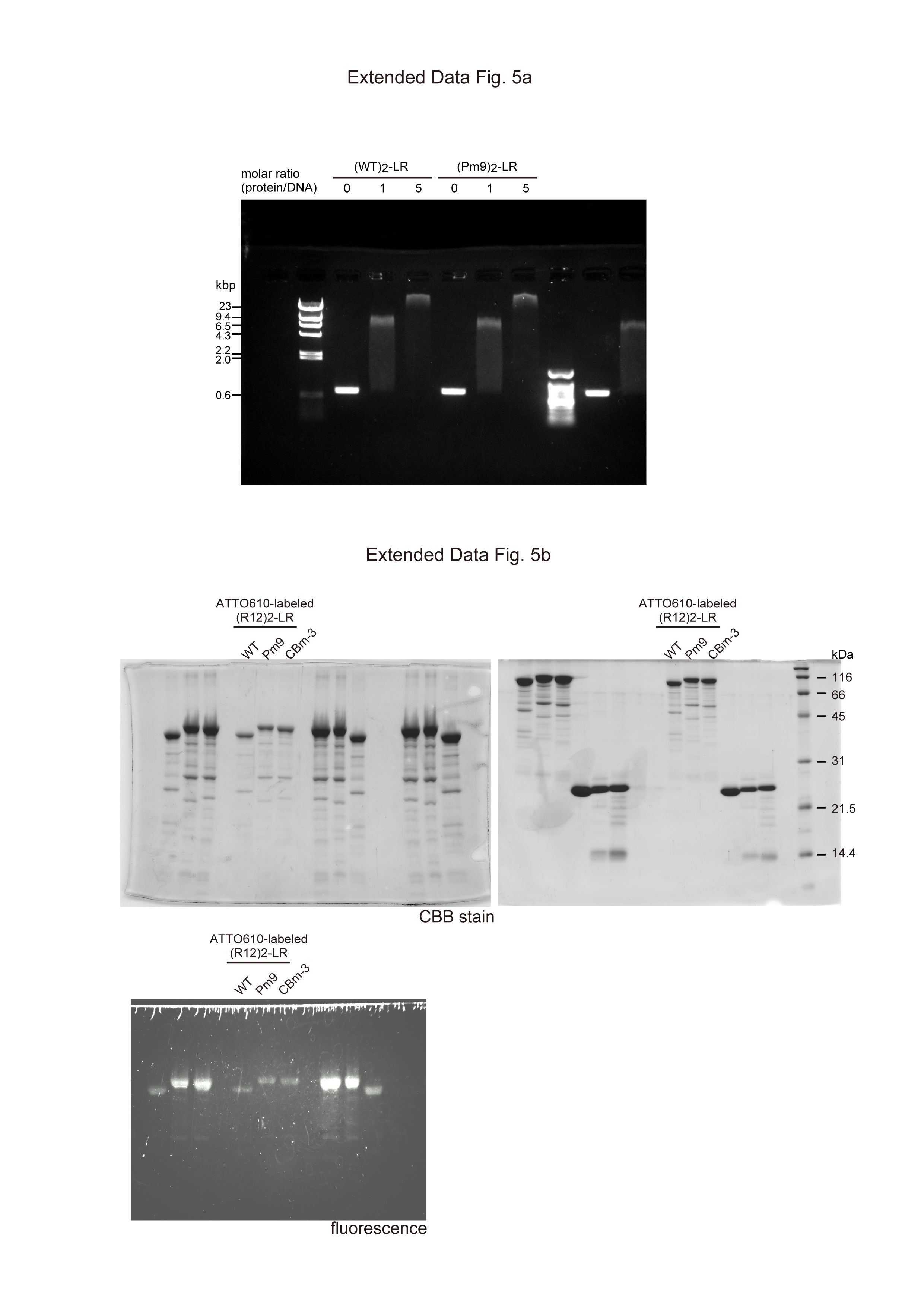

Supplement: Source Data Extended Data Fig. 5 — Unprocessed gels [file 41556_2022_903_MOESM12_ESM.tif]

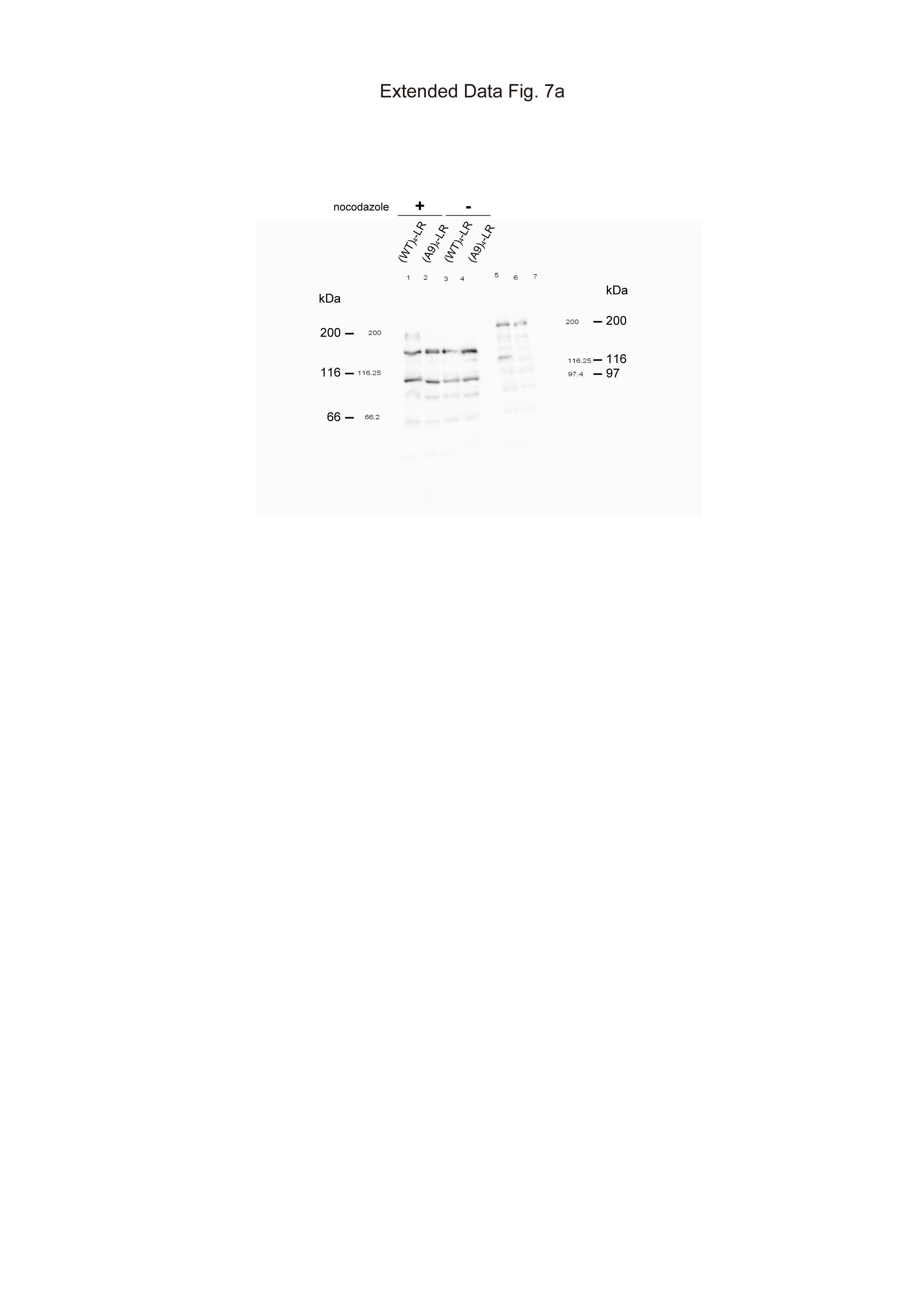

Supplement: Source Data Extended Data Fig. 7 — Unprocessed Western Blot [file 41556_2022_903_MOESM14_ESM.tif]

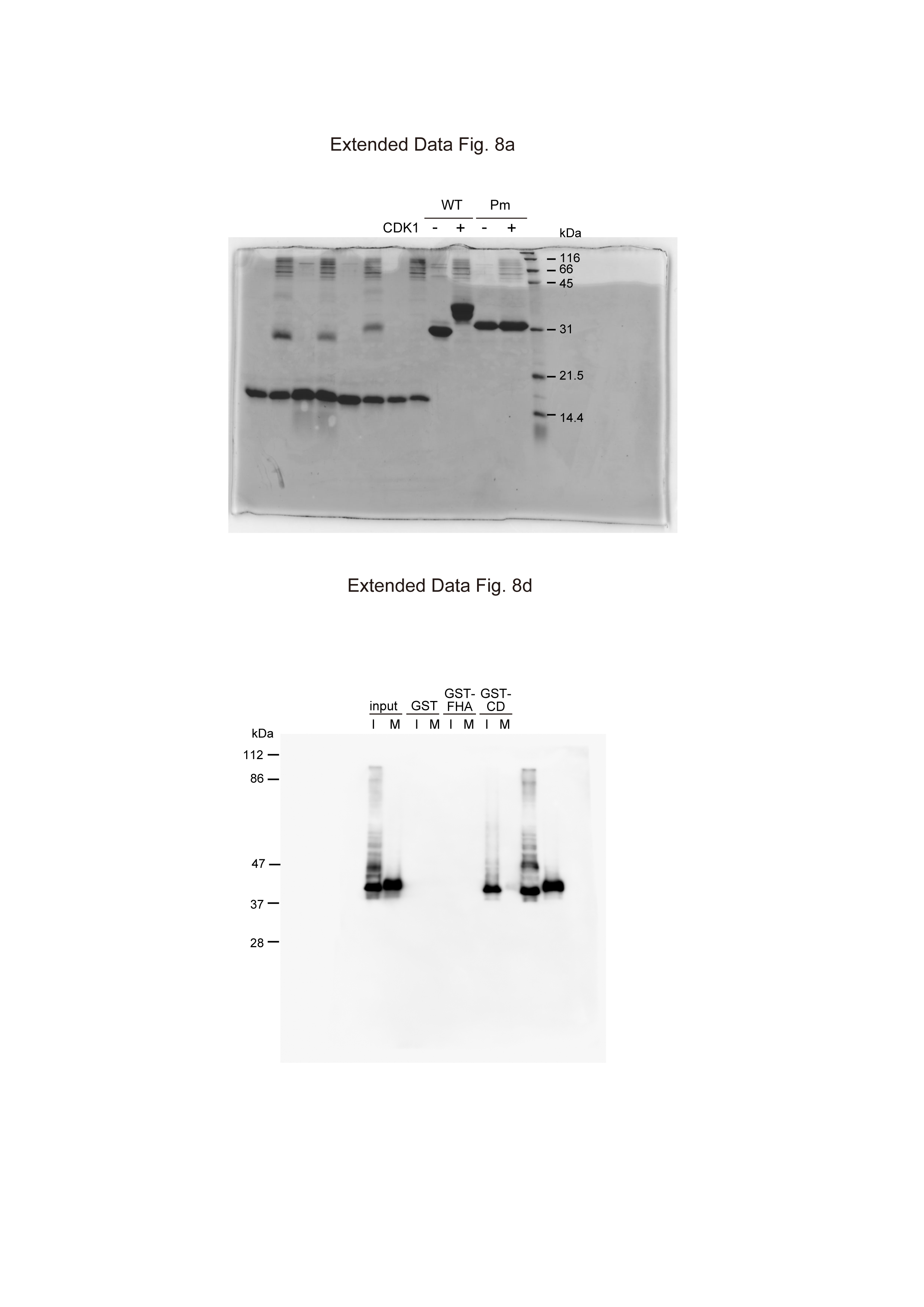

Supplement: Source Data Extended Data Fig. 8 — Unprocessed Western Blot and gel [file 41556_2022_903_MOESM16_ESM.tif]
